# Treatment of Localized and Referred Masticatory Myofascial Pain with Botulinum Toxin Injection

**DOI:** 10.3390/toxins13010006

**Published:** 2020-12-23

**Authors:** Jose-Francisco Montes-Carmona, Luis-Miguel Gonzalez-Perez, Pedro Infante-Cossio

**Affiliations:** 1 Department of Oral and Maxillofacial Surgery, Virgen del Rocio University Hospital, 41013 Seville, Spain; josmoncar@gmail.com (J.-F.M.-C.); pinfante@us.es (P.I.-C.); 2 Department of Surgery, School of Medicine, University of Seville, 41009 Seville, Spain

**Keywords:** temporomandibular disorders, masticatory myofascial pain syndrome, botulinum toxin, randomized controlled trial

## Abstract

Botulinum toxin type A (BTA) injection is considered an available alternative treatment for myofascial pain. However, its efficacy in treating masticatory myofascial pain syndrome (MMPS) remains unclear. The purpose of this study was to evaluate whether the BTA injection into the affected muscles would significantly reduce pain and improve function, and to assess its efficacy, safety, and therapeutic indications in a randomized, single-center clinical trial. Sixty patients with MMPS were randomized into three groups evenly to receive a single session injection of saline solution (SS group), lidocaine (LD group), and BTA (BTA group) in the masseter, temporal, and pterygoid muscles after an electromyographic study. Patients’ pain was classified as localized or referred according to the DC/TMD classification. Assessments were performed on pre-treatment, and subsequently, on days 7, 14, 28, 60, 90, and 180. A significant reduction in pain and improvement of mandibular movements was found in the BTA group compared to the SS and LD groups. The response lasted until day 180 and was more intense in patients with localized myalgia and focused myofascial pain than in referred remote pain. No significant adverse reactions were observed. A single BTA injection can be considered an effective treatment option in patients with localized MMPS by reducing pain and improving mandibular movements, which persisted up to 6 months.

## 1. Introduction

Myofascial pain syndrome is a complex disorder of the musculoskeletal system with multifactorial involvement and diverse clinical presentations in several areas of the body [1]. It can affect the oro-cranio-facial region, with involvement of the temporomandibular area and masticatory muscles, and is then called masticatory myofascial pain syndrome (MMPS). A prime feature of the condition is the existence of trigger points (TrPs), which are areas within the affected muscles that, when stimulated by pressure, cause muscle pain locally or a transfer of pain through radiation to nearby areas of the craniofacial anatomy such as teeth or temporo-mandibular joint (TMJ), producing what is known as referred pain [2]. Some parafunctional habits, such as bruxism, have a significant influence in the pathogenesis and perpetuation of this syndrome. Most opinions agree that abnormal muscle contraction patterns are largely responsible for sustaining the muscle pain [3].

In 2014, the National Institute for Dental and Craniofacial Research revised the Research Diagnostic Criteria for Temporo-Mandibular Disorders (RDC/TMD), thus giving rise to a new classification known as Diagnostic Criteria for Temporo-Mandibular Disorders (DC/TMD) [4,5]. In the DC/TMD classification, pain is a consistent diagnostic criterion, gaining value as the central axis of temporomandibular disorders. Some terms in the RDC/TMD were eliminated, while others such as local myalgia or myofascial pain were included, which would encompass what was previously known as myofascial pain syndrome. This means that the DC/TMD definition contains two possible entities, depending on whether the pain is localized or referred, and the term myalgia describes a pain originating in muscle and which is aggravated with functional or parafunctional movements. According to the DC/TMD classification, the different types of myalgia are fundamentally differentiated by their extension, though maintaining the general characteristics described. Local or localized myalgia (LM) is characterized by pain of muscular origin, but it is localized to the area of palpation on examination. In myofascial pain (MP), soreness and discomfort also originate in the muscle, as in myalgia, but extend to the limits of the considered muscle, beyond the area identified by palpation. Finally, referred myofascial pain (RP) is characterized by extension of the soreness and discomfort towards areas distant from the area identified in the examination and from the limits of the palpated muscle [6].

MMPS is a one of the main reasons for consultation in maxillofacial surgery clinics [7]. Irrespective of whether the condition is acute or chronic, it causes limitations in basic aspects of daily life patients and has severe family and socio-occupational repercussions owing to its effects on mental wellbeing, patient quality of life, and limiting workplace performance. Despite its high prevalence, there is no general agreement concerning its treatment, as it often responds poorly to therapeutic approaches based on occlusion splints, physiotherapy, analgesia, anti-inflammatory, or muscle relaxants [8]. Cases that are refractory to these conservative therapeutic approaches serve as the motivation to seek other treatments.

Botulinum toxin type A (BTA) is included within the available therapeutic array in these refractory cases. BTA has been used for decades in disorders caused by muscle hyperactivity such as strabismus, blepharospasm, spasmodic torticollis, hemifacial spasm, chronic migraine, or chronic musculoskeletal pain [9,10]. It is injected into the affected muscles and acts by chemical denervation and muscle relaxation, thereby breaking the vicious cycle of the muscle hyperactivity-pain [11]. At present, insufficient data are available to definitively assess the beneficial effects of BTA in myofascial syndromes in the mid-term, and scientific evidence is lacking for its therapeutic use to be recommended in cases of refractory myofascial pain with masticatory muscle hyperactivity [12,13]. Studies are, therefore, required to enable guidelines to be established on its proper use and to avoid over-treatment.

Given that scientific evidence justifying the use of BTA is based on the treatment of muscle hyperactivity pathology in other anatomical areas, we chose to review and update what is known about this condition and then study the efficacy, safety, and therapeutic use of botulinum toxin in refractory myofascial syndrome with trigger points in masticatory muscles. 

## 2. Results

**Figure 1:**
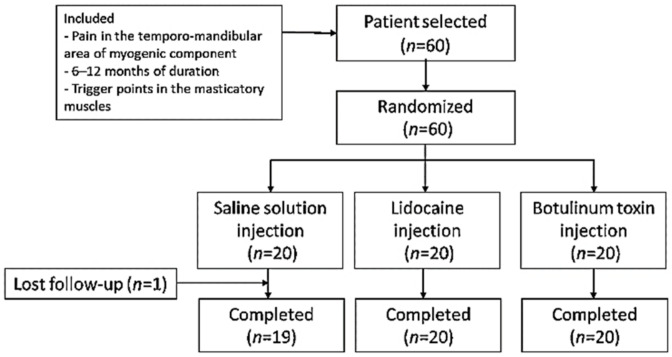
Study flowchart.

Sixty patients were included and randomized to study groups (*n* = 20 in each group) (Figure 1). The three groups presented similar mean age distributions (saline solution (SS) group = 42.95 years, range = 35.94–49.96; lidocaine (LD) group = 45.40 years, range = 38.64–52.16; BTA group = 42.40 years, range 37.21–47.59). There was a predominance of women as evidenced by the female/male ratios for the different groups (SS group = 17/3; LD group = 16/4; BTA group = 16/4). According to the DC/TMD classification, 20/60 (33.3%) patients suffered LM, 27/60 (45%) patients MP, and 13/60 (21.7%) patients RP. The distribution in each study group was SS group (six patients with LM, 10 with MP and four with RP), LD group (seven patients with LM, eight with MP and five with RP) and group BTA (seven patients with LM, nine with MP and four with RP). No significant differences were found between the patient characteristics of the three groups. All patients in the LD and BTA groups completed the trial, while in the SS group, one patient left the study prematurely.

**Figure 2:**
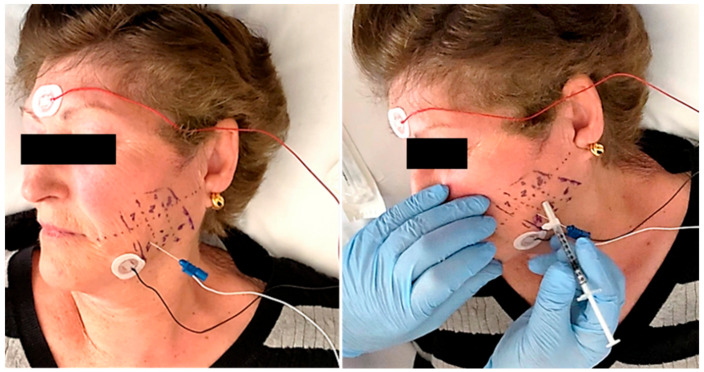
A previous electromyographic study was carried out to detect specific patterns of muscle involvement (**left**). The needle was inserted into the muscle under electromyographic check and the injection was carried out following the study protocol (**right**).

Panoramic radiography and magnetic resonance imaging (MRI) did not reveal significant changes. All patients showed normal electromyographic studies (EMG), but 10 patients (77%) in RP subgroup of three treatments groups (three in SS group, four in LD group, and three in BTA group) showed the presence of myokymic records (regular groups of 2–10 potentials of motor units discharging at 0.1–10 Hz and recurring regularly at 0.2–1 s intervals in doublets or triplets). These patterns were clearly defined after the clinical examination and the EMG study that was performed before injections. This focal myokymia pattern was not observed in the other subgroups. No pathological spontaneous activity, such as fibrillation potentials or complex repetitive discharges, was detected in any patient (Figure 2).

Table 1 shows the results of the intragroup analyses found for the three groups of the study on different days post-therapy, expressed as the mean (M) ± standard deviation (SD). From day 0 to day 180, changes in pain intensity values, maximum interincisal opening (MIO), and right and left lateral and protrusion movements were statistically significant for the BTA group with respect to day 0, but not for the SS and LD groups. The comparative analysis between consecutive checkpoints for the three study groups found significant differences for the BTA group in pain intensity reduction in visual analog scale (VAS) scores (between day 0 and 28) and lateral movements (between day 0 and 7) indicating that the improvement was detected from the first week (Table 2). 

The analysis of the differences between groups in consecutive checkpoints is shown in Table 3. Significant variations in pain intensity were found for the BTA group versus SS group from day 14 and versus LD group from day 7, which was maintained until the end of the study, indicating that the improvement was prolonged. 

The analysis of the intragroup differences according to the classification of localized or referred muscle pain is shown in Table 4. In the BTA group, a significant improvement was found in both localized and referred pain until day 180, unlike the SS and LD groups that did not display statistical significance. In the analysis according to the classification of localized or referred muscle pain, significant intragroup variations were found for the BTA group when comparing RP subgroup with LM subgroup and MP subgroup from day 14 to day 180, signifying that pain relief was significant and prolonged in the mid-term in patients with localized muscle pain (Table 5).

**Figure 3:**
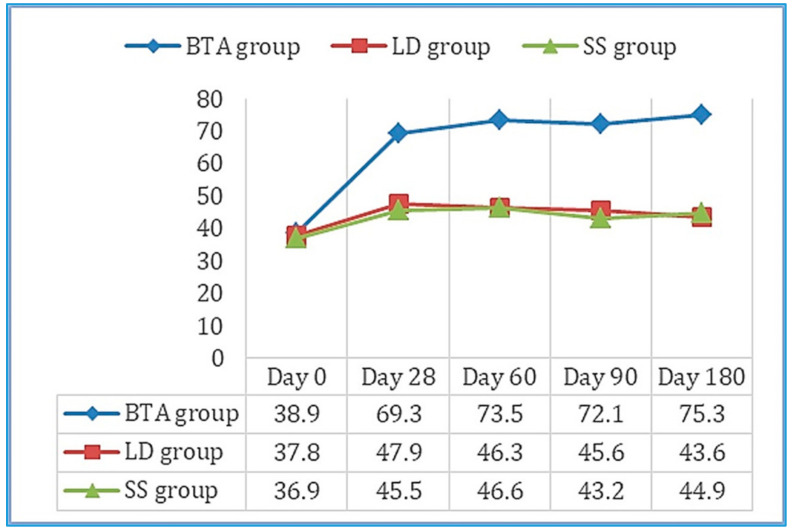
Mean scores obtained on the 100-point questionnaire throughout the follow-up period in the three study groups. Results improved significantly between day 0 and 180 in the BTA group.

Values obtained in the 100-point questionnaire improved significantly between day 0 and day 180 in the BTA group (Figure 3), as well as the evaluation of the efficacy outcomes and tolerance both for the patient and the observer. Six patients reported mild side effects, two in each group, which consisted of pain (three cases: SS group—one with MP and one with RP—and BTA group—one with RP), hematomas in the puncture area (two cases: LD group—one with LM—and BTA group—one with LM), and headache (one case: LD group with RP). None of these adverse effects prevented the patients’ participation in the study.

## 3. Discussion

Various therapeutic procedures are available for the management of chronic muscle pain, although no specific treatment has proven to be consistently effective. The primary mode of action of conservative treatments lies in reducing muscle tone and achieving relaxation. In this way, a large percentage of cases can obtain a satisfactory reduction in pain after following one or more conservative treatment modalities. There remains, however, a significant group of refractory cases that must be therapeutically oriented towards minimally invasive treatments, among which are the puncture and injection of different therapeutic substances [8].

MMPS is a common pain syndrome and a comorbidity complicating other diseases. It is characterized by TrPs that are areas of intense tenderness located on a contracted band of masticatory muscles. The local twitch response is a characteristic finding of MMPS; it is activated by pressure, palpation, or needle insertion at TrPs and is manifested by a burst of activity in the muscle band that contains the activated point. Treatment of MMPS involves the inactivation of TrPs. In many cases, repeated inactivation generally reverses central sensitization, so that TrPs no longer form repeatedly. However, in some patients, these points continue to recur despite repeated inactivation. In these patients, additional steps must be taken to inactivate TrPs and prevent their recurrence. It is in these situations that BTA could play an effective role, as it occurred in the patients included in our study, because of its long duration of action. In these cases, BTA decreases the electrical activity of the TrPs and, therefore, should eliminate the taut band that underlies the TrP [2,7,14]. 

The neurophysiological mechanisms involved in MMPS are difficult to resolve from clinical studies because of the great variability among patients, which may be originated by different factors such as pain intensity or location of muscle pain. There are different theories that have been proposed to explain the findings observed in TrPs: muscle spindle hyperactivity theory (TrPs are at the site of the muscle spindle, and the increased activity at this location is directly associated with hyperactivity of muscle spindle), end-plate hyperactivity theory (when TrPs is due to hyperexcitable motor end-plates), and focal dystonia theory (when the activity in a TrP is depending on a focal dystonia), but no single theory completely explain all the known data concerning TrPs and further research is needed to more accurately understand these neurophysiological findings [2,7,14]. 

BTA is a relatively recent treatment for MMPS, but few scientific studies support its use, clearly due to the considerable heterogeneity of the populations studied so far [15,16,17,18,19,20,21,22,23]. BTA interferes muscle contraction by preventing the release of acetylcholine from motor nerve endings at the neuromuscular junction and have an analgesic effect on nociception mediated by an inhibition of neurotransmitter release from peripheral nociceptors that is quite separate from its effect on acetylcholine release in motor nerve terminal. The benefits of the muscle relaxant and analgesic properties of BTA were first shown by Freund and Schwartz [24], who reported that 90% of patients with cervicofacial muscle soreness and discomfort showed improvements in pain and function after the local application of BTA [25]. Subsequently, several case series and cohort studies have suggested different dilutions, techniques, and doses of BTA and have also shown an improvement in the symptoms of temporo-mandibular disorders [21].

It seems clear, however, that despite several clinical trials the myofascial pain relief efficacy of the botulinum toxin has not yet been established [26]. We consider that the main basis for this discrepancy is related to the large differences in clinical diagnostic criteria and in inclusion and exclusion criteria used in the different trials that have been analyzed. As various diagnostic criteria exist for myofascial pain, patients included in some studies might have been excluded from others. This could cause the effect size to be underestimated, due to the inclusion of patients unlikely to respond to treatment and thereby giving the misleading appearance of a failed trial when, in fact, a sub-group of the study may have experienced real benefits [15,16,17,18,19,20,21,22,23,24,25,26].

In our study, we observed a clear improvement in refractory MMPS, with a significant reduction in pain in the masticatory muscles following the injection of BTA in cases of LM and MP. This positive response, although significant, was not seen with to the same degree in patients with referred myofascial pain with irradiation to areas distant from the masticatory muscles (RP). In addition, in the sub-group of patients with referred pain who received BTA treatment, we observed a slower response in the appearance of reductions in VAS from the first month, while in the sub-groups with localized pain (LM and MP groups), the reduction was already significant after 7 days in 55% of cases and remained stable throughout the study follow-up. In our opinion, the differential aspects of RP are based on central mechanisms, and central sensitization may be involved in the origin of RP; therefore, their management and therapeutic approach must be different. Consequently, it is helpful to use consistent diagnostic criteria for temporo-mandibular disorders and to classify patients with refractory MMPS into subgroups according to pain type and location. This would enable BTA to be used specifically in those patients in whom a better response is expected. 

As for the other two groups in the study, the first (SS) was clearly a placebo group, since the content of the syringes was saline. For the second group receiving lidocaine (LD), we assumed that the peripheral block can be effective in some patients with facial pain disorders; pain suppression in these cases is based on the therapeutic effect of low concentrations of local anesthetics to selectively block sensory fibers in mixed nerves. In our experience, this can be useful in the differential diagnosis of referred pain conditions. Various authors consider that motor function is preserved or minimally affected in these patients. Of course, the duration of the block depends on the dose and the pharmacokinetics of the anesthetic used, but in clinical practice, a longer than expected duration of benefits is observed in many cases [27,28]. However, no therapeutic response was seen in the LD group of our study from the first post-injection follow-up, an observation that persisted for the duration of the study. Our study had a low dropout rate in the placebo group with saline, with the loss of only one patient due to a change of address. The other two groups, LD and BTA, did not have any dropouts, and all patients completed the follow-up visits. We believe that the key to obtaining this high adherence rate was the motivation of the patients included in our study [22].

There are studies that favor the use of BTA, such as the one by Sidebottom et al. [29] who conducted a 6-week prospective study with 62 patients. A positive response (VAS pain reduction > 25%) was seen in 79% of the cases, with the injection of BTA determined to be a useful treatment within the therapeutic options for refractory MMPS, despite complete resolution not being obtained. Patients in the different groups in our trial were of a similar mean age, gender distribution, and identified pain patterns. Furthermore, we believe that the recording of EMG activity prior to performing the treatment is useful to identify the muscle masses to be treated and to avoid side effects. One hundred and eighty days after the injection of BTA, the different scores obtained in our cases showed a significant reduction in pain in all patients, with a total mean pain reduction in VAS scores from 6.5 ± 0.94 to 2.95 ± 1.05 (*p* = 0.001), including localized pain in its two varieties (from seven to two in the LM subgroup, and from six to two in the MP subgroup). The reduction, although significant, was not as evident in patients with RP in the BTA group (pain reduction from 6.5 to 4 in VAS).

Retrospective studies, such as that by Stonehouse-Smith et al. [30], which included 100 patients with temporo-mandibular myofascial pain, found a pain reduction of 2.48 points on the VAS scale 16 weeks after the injection of BTA in doses of 100 units. No significant differences in pain reduction were seen in patients injected with doses of 200 units (20). In our bibliographic review, we also found a considerable variation in the masticatory muscles injected and in the doses of BTA used. All studies reported injection into the masseter muscle, but with a wide variability in the other muscles treated. For example, Emberg et al. [23] treated the masseter muscle alone, possibly limiting the observable effects, while the other studies reported the injection of at least one temporal muscle along with the masseter injection, with Lindern et al. including the medial pterygoid [21] and Nixdorf et al. the lateral pterygoid [22]. The total dose ranged from 50 units unilaterally to 300 units bilaterally [30]. In our trial, doses between 100 and 150 units bilaterally distributed to the masseter, temporal, and pterygoid muscles were used.

We also found variations in the designs of the analyzed studies, inconsistent reports on assessment tools, and heterogeneous study groups [15,16,17,18,19,20,21,22,23,24,25,26,31]. Overall, the level of bias we found was moderate to high, making the quality of the scientific evidence moderate to low. Although benefits derived from the use of BTA were observed in various reports, a clear consensus on the therapeutic benefit of BTA in the treatment of MMPS was not present, meaning that additional randomized clinical trials with minimal bias, larger samples, and longer follow-up periods should be undertaken. The optimum location to inject the BTA and the optimal dose must also be established, in addition to which feasibility studies on the cost of BTA treatment compared to other therapeutic options should be performed and the cost-benefit ratio assessed for its clinical acceptability. Most of the analyzed studies had small samples (around 10–14 patients per group), which has ultimately reduced the quality of the scientific evidence. We estimated that a sample size for such studies needs to be 20 per group. This value was estimated from the required change in VAS scores (approximately 30%) and the existing standard deviations of previously reported results [21,32,33,34].

The onset of pain reduction with BTA is established in a delayed manner after its administration, which in our study, was significant after 7 days, especially in patients with a localized pain pattern, probably due to the analgesic effect of the toxin that accumulates gradually, reaching a peak of effectiveness after a week of continuous muscle relaxation. Similar to the more common conservative treatment modalities, the analgesic effect of BTA appears after a period of continuous and sustained relaxation of pain generated by fatigued muscle fibers. The effect of BTA on MMPS lasts for at least 6 months in duly selected cases, since in our study, most patients in the BTA group reported at the last check-up that they had not reverted to pain levels experienced prior to the administration of BTA. 

We did not observe a reduction in normal mandibular mobility figures in patients prior to starting our trial, and therefore, the figures obtained after treatment showed few changes except in the BTA group in which a significant improvement in mandibular function was found in maximum interincisal opening (MIO) and laterality and protrusive movements (Table 1). From the start of the study until day 180, pain decreased significantly from 6.5 (±0.94) to 2.95 (±1.05). The intragroup comparative analysis for every two consecutive control points showed significant improvements for pain up to day 28 (Table 2), suggesting that pain relief was more evident during the first month, but its effect was continued until 6 months. In our experience, limited mouth opening is usually a common complaint in three categories of temporo-mandibular disease: displacement of the articular disc, with or without reduction, and advanced degenerative osteoarthritis. Forms of myofascial pain without concomitant joint involvement, such as those analyzed in our study, can retain normal functional mobility. This, however, was not reported as part of the diagnosis in any of the available studies. Although several publications have reported contradictory results, Emberg et al. [23] found no significant changes with initial measurements of MIO between 43.0 and 46.3 mm, taking into account that normal MIO is 51.3 ± 8.3 mm in men and 44.3 ± 6.7 mm in women, and that this MIO undergoes a reduction with age. Based on current evidence, no definitive conclusions can be drawn as to whether BTA injections improve oral opening. It would be useful to conduct studies to compare the results of BTA injections in patients with a primary complaint of limitation of oral opening compared to the administration of placebo, which will be the topic of future studies.

Finally, all of our patients were evaluated concerning the known contraindications for BTA. All adverse effects experienced by patients were temporary and included localized pain, hematoma, and transient headache. We did not observe in our study any form of paralysis of the zygomaticus major muscle, which is characterized as an asymmetric smile, common in other studies. We believe this may be the result of the local diffusion of BTA from the masseter muscle, or due to direct trauma to the muscle aggravated by the usual practice of massaging the injected area. We consider it necessary to avoid this procedure. It is important to consider that the local diffusion of BTA may depend on the dose and the volume used, since large volumes can compromise the integrity of the muscle fasciae, which is another point that must be taken into account to minimize side effects in future studies. The high costs of BTA treatment compared to other conservative measures must also be considered. In a pilot study which analyzed the osteopenic consequences of BTA injections in the masticatory muscles, Raphael et al. found a reduced bone density in all patients who had been exposed to BTA, and a normal density in those who had not [15]. In our cases, despite not having performed bone densitometry studies, we did not observe any loss of bone density in a follow up analysis of radiological records. 

This study has some limitations. Treatment review was limited to the effects seen in the mid-term. A study with a larger size sample and a longer follow-up period is required to determine the long-term benefits of BTA injection into masticatory muscles. To improve the validity of the study, it would be interesting to assess the treatment in patients with fibromyalgia or depression, which in this study were excluded.

## 4. Conclusions

This six-month prospective study has evaluated the efficacy of BTA in the treatment of refractory MMPS and differentiated between two detected patterns of myofascial pain: localized myofascial pain and non-localized, radiated or referred myofascial pain. In our study, these patterns were clearly defined after the clinical examination and the EMG study performed prior to injections. The results obtained show a significant reduction in pain following the injection of BTA in all patients with localized refractory masticatory myofascial pain, which persisted up to 6 months, in its two varieties (LM and MP groups). A decrease in pain intensity, although significant, did not reach very low values in any of the patients with referred pain (RP group). BTA should be thus considered as a safe therapeutic option in patients with localized masticatory myofascial pain where a better response to treatment is expected, and where it has been shown to be more effective. 

## 5. Materials and Methods

### 5.1. Standard Protocol Approvals and Patient Consents

This study protocol was approved by the Institutional Review Board (IRB) of the Virgen del Rocio University Hospital (IRB number: 2013PI/119) with approval date on 7 January 2014, and guidelines of the Declaration of Helsinki were followed. All participants were informed of the nature of the study, and written informed consent was provided by all the subjects participated in the study.

### 5.2. Study Design and Subjects

A randomized, single-center clinical trial was conducted between July 2015 and June 2016. Patients of both genders, between 18 and 75 years of age, with myofascial pain in the temporo-mandibular area, were recruited after ruling out the presence of other pathologies by means of a panoramic radiography and MRI study. Patients with pain in the temporo-mandibular area with a myogenic component of 6 to 12 months of evolution, without severe limitation in joint mobility or signs of internal damage, and with trigger points in the masticatory muscles were included. Individuals were excluded if they presented one or more of the following conditions: concomitant treatments with aminoglycosides or quinolones, inflammatory processes at the proposed injection area, pregnancy, lactation, internal damage to the TMJ, degenerative joint pathology, dentofacial deformities, previous jaw trauma, chronic degenerative neuromuscular disorders, increased bleeding tendency or if taking anticoagulants, tension or migraine headaches, an infectious-inflammatory history of odontogenic origin, belonephobia, fibromyalgia, uncontrolled metabolic disease, or significant depression. 

A baseline clinical examination was performed to determine if the pain was of joint or muscle origin. If it was muscle-derived pain, it was classified as localized or referred pain according to the DC/TMD classification [2,3], as (1) localized myalgia (LM): myogenic pain located in a hypersensitive nodule; (2) myofascial pain (MP): localized myalgia extending within the limits of the affected muscle; (3) referred pain (RP): when pain extends to areas outside the limits of the palpated muscle. In our cases, the palpation of an indurated and hypersensitive nodule, within a musculature of normal consistency, was the physical finding typically associated with a trigger point. In LM and MP cases, the palpation caused pain directly in the affected area, whereas in RP cases, pain radiated to a reference area, such as the posterior teeth, the TMJ, or the cervical region. 

Examination of the masticatory musculature focused on the masseter, temporal, medial pterygoid and lateral pterygoid muscles, bilaterally, combining pressure, clamping, and sliding movements to detect the existence of trigger points and areas of muscle pain. The masseter was examined by extra- and intraoral palpation, inside the cheek and outside, along the lower face of the zygomatic arch, and following a union line between the mandibular angle and the nasal ala. The temporal muscle examination was made externally from behind the outer canthus of the eye to behind the external auditory canal, including the intraoral palpation of the tendon insertion over the coronoid process. The medial pterygoid was palpated intraorally behind the last molar and extraorally at the mandibular basal insertion. The lateral pterygoid was assessed intraorally behind the maxillary tuberosity and with protrusive movement against resistance. An EMG study was performed before the injections in order to identify the muscle masses to be treated and to detect specific patterns of normality or muscle involvement, at rest and during voluntary activity. The equipment we used was a 10 channel Medelec Synergy, Oxford Instruments, Abingdon, Oxfordshire, UK (computer version 22.1.1.153, software 2016). 

Patients were randomly assigned by a random number generator to one of three treatment groups: SS, LD, and BTA. The treatments were performed in a single session to allow an assessment of the efficacy and safety of the treatment and its therapeutic indications. Clinical evaluations were performed on the pretreatment baseline day (day 0), and subsequently on days 7, 14, 28, 60, 90, and 180. Data were collected at each visit by the same observer. The injection protocol was identical for the three groups: (1) Manual localization of the muscle mass and intramuscular puncture-injection after the EMG study. (2) Selection of 1 mL BD U-100 insulin syringes with a decimal scale graduation and fitted with 30G needles of 13 mm length. (3) Injection material: for the SS group, an injectable 0.9% saline solution; for the LD group, 2% lidocaine with vasoconstrictor; for the BTA group: onabotulinumtoxin A (Botox^®^), where 50 units were diluted in 1.25 mL of saline to obtain 4 units of BTA for every 0.1 mL of injection fluid. The administered dose was 100–150 units distributed among the different muscle masses bilaterally. (4) Prior to muscle puncture, the area was cleaned with 90° alcohol. The puncture was performed manually by pinching the overlying skin with 2 fingers. The injection was performed at 3 points in the temporal muscle (total amount of 0.2 × 3 = 0.6 mL), 3 points in the masseter muscle ((0.2–0.25) × 3 = 0.6–0.75 mL), 1 point in the lateral pterygoid muscle (0.2 mL), and 1 point in the medial pterygoid muscle (0.2 mL) on each side; therefore, taking into account the dilution used in the BTA group, injecting doses of 8 units × 3 = 24 units in the temporal muscle, 8–10 units × 3 = 24–30 units in the masseter muscle, 8 units × 1 = 8 units in the lateral pterygoid muscle and 8 units × 1 = 8 units in the medial pterygoid muscle, bilaterally. After the injection, hemostatic compression was applied for one minute. No muscle stretching was performed after the injection. 

### 5.3. Measurements

The main parameters used to evaluate the effectiveness of the treatment were: (1) Pain at rest and when chewing, assessed with a visual analog scale (VAS, 10 cm) and (2) range of mandibular movements associated with the opening of the mouth, lateral, and protrusion movements measured with a Therabite^®^ ruler (Atos Medical, New Berlin, WI, USA). Signs that were evaluated as indicators of effectiveness were significant reduction in myofascial pain at rest and with mastication, recovery of normal ranges of mandibular opening, lateral and protrusive movements, and improvement in TMJ function. Affectation of the TMJ was evaluated via a questionnaire consisting of a scale of 100 points (being 0 the worst, 100 the best), assessing pain (40 points), function (45 points), and chewing (15 points) [2,8]. Secondary efficacy outcomes were overall efficacy assessments estimated by the patient and investigators using a 5-point scale ranging from 0 (worst) to 4 (optimal). Tolerance to the treatment was assessed by patients and investigators, using a 5-point scale (0—very bad, 1—bad, 2—fair, 3—good, 4—excellent). The type and frequency of adverse events were recorded at each visit. 

### 5.4. Statistical Analysis 

Data were analyzed with the IBM SPSS Statistics 20.0.1 (IBM Co., Armonk, New York, USA), software 2012. The sample size was estimated with a view to identifying a decrease of 2 or more pain points in the visual analog scale (VAS) after the injection of BTA that should be present within 2 weeks of the procedure. Considering 5% as the significance level and 1.35 as the equivalence limit, we performed a triple unilateral equivalence Student’s *t*-test based on 3 independent series, which gave us 19 necessary cases per tested group. By estimating a possible dropout rate of 2%, the recruitment of 20 individuals was considered necessary for each group undergoing the different therapeutic procedures (*n* = 60 patients total). The data were first analyzed with a general statistical test (Friedman’s test), using absolute and relative frequencies in the case of qualitative variables and mean values, standard deviation (SD), or the 50th percentile (P50; median = Me), or P25–P75 interquartile ranges (IQR) for quantitative variables. Comparisons between the three groups were made with the Kruskal-Wallis test for each checkpoint. Before and after comparisons of variables in each group were made with Friedman’s test, while within group differences were analyzed with the Wilcoxon signed rank test (with post hoc Bonferroni correction) to analyze intragroup variations. When differences between the three groups were detected, the Mann-Whitney U test was used to analyze between two groups which groups differed from each other (with post hoc Bonferroni correction). Values of *p* < 0.05 were considered to indicate statistical significance. 

## Figures and Tables

**Table 1:** Intragroup analysis at each checkpoint.

Group	Day 0	Day 7	Day 14	Day 28	Day 60	Day 90	Day 180	
	M (± SD)	M (± SD)	M (± SD)	M (± SD)	M (± SD)	M (± SD)	M (± SE)	*p*-Value
Pain (VAS)								
SS	6.47 (± 0.96)	5.42 (± 1.16)	5.47 (± 1.26)	5.57 (± 1.30)	5.78 (± 1.18)	6 (± 1)	6 (± 0.94)	−
LD	6.45 (± 1.09)	6.55 (± 1.05)	6.4 (± 1.14)	6.5 (± 1.27)	6.3 (± 0.80)	6.5 (± 1.05)	6.5 (± 1)	−
BTA	6.5 (± 0.94)	4.95 (± 1.27)	3.6 (± 1.53)	3 (± 1.41)	2.75 (± 1.16)	2.85 (± 0.93)	2.95 (± 1.05)	< 0.0001 **
Maximum interincisal opening (mm)								
SS	43.05 (± 6.88)	43.05 (± 7.09)	43.33 (± 6.76)	43.05 (± 6.88)	43.05 (± 6.88)	43.05 (± 6.88)	43.05 (± 6.88)	0.287
LD	43.35 (± 5.18)	43.25 (± 4.78)	43.2 (± 5.11)	43.4 (± 5.17)	43.5 (±5.29)	43.2 (± 4.96)	43.4 (± 5.15)	0.4
BTA	40.7 (± 5.43)	41.55 (± 5.15)	42.1 (± 5.43)	42.3 (± 5.35)	43.15 (± 5.19)	43.4 (± 5.19)	43.4 (± 5.19)	< 0.0001 **
Lateral—Right (mm)								
SS	5.88 (± 1.56)	6 (± 1.57)	5.88 (± 1.45)	5.88 (± 1.52)	5.94 (± 1.62)	5.94 (± 1.62)	5.94 (± 1.62)	0.774
LD	5.55 (± 1.63)	5.6 (± 1.56)	5.6 (± 1.56)	5.7 (± 1.65)	5.6 (± 1.63)	5.65 (± 1.66)	5.55 (± 1.53)	0.167
BTA	5.8 (± 1.70)	6.25 (± 1.86)	6.6(± 1.87)	6.85 (± 1.72)	6.7 (± 1.78)	6.6 (± 1.63)	6.45 (± 1.60)	< 0.0001 **
Lateral—Left (mm)								
SS	5.94 (± 1.62)	5.94 (± 1.51)	5.88 (± 1.45)	5.83 (± 1.46)	5.88 (± 1.52)	5.94 (± 1.62)	5.88 (± 1.56)	0.811
LD	5.55 (± 1.63)	5.6 (± 1.56)	5.6 (± 1.56)	5.7 (± 1.65)	5.55 (± 1.63)	5.6 (± 1.63)	5.55 (± 1.53)	0.609
BTA	5.8 (± 1.70)	6.25 (± 1.86)	6.55 (± 1.87)	6.75 (± 1.71)	6.65 (± 1.78)	6.55 (± 1.63)	6.45 (± 1.60)	< 0.0001 **
Protrusion (mm)								
SS	4.77 (± 0.87)	4.77 (± 0.87)	4.83 (± 0.92)	4.83 (± 0.98)	4.83 (± 0.98)	4.83 (± 0.98)	4.83 (± 0.98)	0.809
LD	4.7 (± 0.86)	4.7 (± 0.86)	4.75 (± 1.01)	4.75 (± 1.01)	4.65 (± 0.81)	4.75 (± 1.01)	4.75 (± 1.01)	0.423
BTA	4.6 (± 1.14)	4.9 (± 1.29)	5.25 (± 1.20)	5.4 (± 1.14)	5.35 (± 1.18)	5.35 (± 1.22)	5.2 (± 1.15)	< 0.0001 **

Abbreviations: M = mean. SD = standard deviation. VAS = visual analog scale. SS = saline solution group. LD = lidocaine group. BTA = botulinum toxin group. Significance (*p*): Friedman test for intragroup comparative analysis. Results were considered highly significant (*p* < 0.001) **.

**Table 2:** Intragroup analysis every two consecutive checkpoints.

Group	Δ 0–7	Δ 7–14	Δ 14–28	Δ 28–60	Δ 60–90	Δ 90–180
	*p*-Value	*p*-Value	*p*-Value	*p*-Value	*p*-Value	*p*-Value
Pain (VAS)						
BTA	< 0.0001 **	0.002 *	0.005 *	0.025	0.317	0.157
Maximum interincisal opening (mm)						
BTA	0.017	0.01	0.588	0.017	0.034	1
Lateral—Right (mm)						
BTA	0.007 *	0.033	0.102	0.257	1	0.18
Lateral—Left (mm)						
BTA	0.007 *	0.035	0.102	0.257	1	0.414
Protrusion (mm)						
BTA	0.02	0.011	0.083	0.317	1	0.083

Abbreviations: Δ = change. VAS = visual analog scale. BTA = botulinum toxin group. Significance (*p*): Wilcoxon test for intragroup comparative analysis every two consecutive checkpoints. Results were considered significant (*p* < 0.0083) * and highly significant (*p* < 0.001) **.

**Table 3:** Intergroup analysis at each checkpoint.

Outcomes	Day 0	Day 7	Day 14	Day 28	Day 60	Day 90	Day 180
	*p*-Value	*p*-Value	*p*-Value	*p*-Value	*p*-Value	*p*-Value	*p*-Value
Pain (VAS)							
*p*-value (1)	0.989	0.001 *	< 0.0001 *	< 0.0001 *	< 0.0001 *	< 0.0001 *	< 0.0001 *
SS vs. LD *p*-value (2)	−	0.016	0.025	0.06	0.159	0.135	0.117
SS vs. BTA *p*-value (2)	−	0.235	0.001 **	< 0.0001 **	< 0.0001 **	< 0.0001 **	< 0.0001 **
LD vs. BTA *p*-value (2)	−	< 0.0001 **	< 0.0001 **	< 0.0001 **	< 0.0001 **	< 0.0001 **	< 0.0001 **
Maximum interincisal opening (mm)							
*p*-value (1)	0.311	0.6	0.805	0.99	0.861	0.581	0.609
Lateral—Right (mm)							
*p*-value (1)	0.811	0.707	0.291	0.148	0.223	0.196	0.229
Lateral—Left (mm)							
*p*-value (1)	0.776	0.728	0.338	0.145	0.198	0.198	0.215
Protrusion (mm)							
*p*-value (1)	0.576	0.738	0.14	0.03 *	0.047 *	0.074	0.143
SS vs. LD *p*-value (2)	−	−	−	0.482	0.548	−	−
SS vs. BTA *p*-value (2)	−	−	−	0.078	0.083	−	−
LD vs. BTA *p*-value (2)	−	−	−	0.01 **	0.019	−	−

Abbreviations: VAS = visual analog scale. SS = saline solution group. LD = lidocaine group. BTA = botulinum toxin group. Significance (*p*) (1): Kruskal–Wallis test for intergroup comparative analysis between the three groups in each checkpoint. * Results considered significant (*p* < 0.05). Significance (*p*) (2): Mann–Whitney test for intergroup comparative analysis between two groups when differences were detected with the Kruskal–Wallis test in each checkpoint. ** Results considered significant (*p* < 0.017).

**Table 4:** Intragroup analysis according to the pattern of localized, non-localized, or referred myofascial pain at each checkpoint.

Outcomes	Day 0	Day 7	Day 14	Day 28	Day 60	Day 90	Day 180	
	Me (IQR)	Me (IQR)	Me (IQR)	Me (IQR)	Me (IQR)	Me (IQR)	Me (IQR)	*p*-Value
SS group								
LM, *n* = 6	6.5 (5.75–7.25)	5.5 (4.75–7)	6 (5.25–7.25)	6 (4.5–7.25)	6.5 (4.75–7.25)	6.5 (5–7.25)	6.5 (5.75–7.25)	–
MP, *n* = 10	6 (6–7.5)	6 (5–6.5)	5 (5–6)	6 (5–6.5)	6 (5–7)	6 (5.5–6.5)	6 (5–6.5)	–
RP, *n* = 4	6 (5–7)	4.5 (3.25–5.75)	5 (3.5–5.75)	5 (3.5–5.75)	5 (4.25–5.75)	5.5 (4.25–6.75)	5.5 (5–6.75)	–
LD group								
LM, *n* = 7	7 (5–7)	7 (6–7)	6 (6–7)	7 (5–7)	6 (6–7)	7 (5–7)	7 (6–7)	0.505
MP, *n* = 7	6 (5.25–6.75)	6 (5.25–7)	6 (5.25–7)	5.5 (5–7.75)	6 (5.25–6.75)	6.5 (5.25–7.75)	6 (5.25–7)	0.283
RP, *n* = 5	8 (6–8)	8 (6–8)	8 (5.5–8)	8 (6.5–8)	7 (6–7)	7 (6–7.5)	7 (6–8)	0.178
BTA group								
LM, *n* = 7	7 (6–7)	5 (4–6)	3 (2–5)	2 (2–2)	2 (2–2)	2 (2–2)	2 (2–2)	< 0.0001 *
MP, *n* = 9	6 (5–7.5)	4 (3.5–5.5)	3 (2–3)	2 (2–3)	2 (2–3)	2 (2–3)	2 (2–3)	< 0.0001 *
RP, *n* = 4	6.5 (6–7)	6 (5–7)	6 (5.25–6.75)	5 (4.25–6.5)	4 (4–5.5)	4 (4–4.75)	4 (4–5.5)	0.024 *

Abbreviations: Me = median. IQR = interquartile range. SS = saline solution group (completed *n* = 19). LD = lidocaine group (completed *n* = 20). BTA = botulinum toxin group (completed *n* = 20). LM = localized myalgia. MP = myofascial pain. RP = referred pain. Significance (*p*): Friedman test for intragroup comparative analysis in each checkpoint. * Results considered significant (*p* < 0.05).

**Table 5:** Intergroup analysis according to the pattern of localized, non-localized, or referred myofascial pain at each checkpoint.

Outcomes	Day 0	Day 7	Day 14	Day 28	Day 60	Day 90	Day 180
	*p*-Value	*p*-Value	*p*-Value	*p*-Value	*p*-Value	*p*-Value	*p*-Value
SS group							
*p*-value (1)	0.621	0.278	0.215	0.318	0.282	0.559	0.335
LD group							
*p*-value (1)	0.202	0.232	0.261	0.146	0.363	0.672	0.267
BTA group							
*p*-value (1)	0.989	0.148	0.008 *	0.004 *	0.006 *	0.008 *	0.008 *
LM vs. MP *p*-value (2)	–	–	0.49	0.301	0.39	0.81	0.514
LM vs. RP *p*-value (2)	–	–	0.012 **	0.005 **	0.004 **	0.006 **	0.006 **
MP vs. RP *p*-value (2)	–	–	0.004 **	0.005 **	0.008 **	0.007 **	0.008 **

Abbreviations: SS = saline solution group. LD = lidocaine group. BTA = botulinum toxin group. LM = localized myalgia. MP = myofascial pain. RP = referred pain. Significance (*p*) (1): Kruskal-Wallis test for intergroup comparative analysis between the three groups in each checkpoint. * Results considered significant (*p* < 0.05). Significance (*p*) (2): Mann-Whitney test for intergroup comparative analysis between two groups when differences were detected with the Kruskal-Wallis test in each checkpoint. ** Results considered significant (*p* < 0.017).
